# Molecular Evaluation of a Case of *Fasciola hepatica* in Wild Boar in Southwestern Iran: A Case Report

**Published:** 2018

**Authors:** Bahador SARKARI, Majid MANSOURI, Shamsi NOORPISHEH GHADIMI, Samaneh ABDOLAHI KHABISI, Abdolla DOSHMANZIARI

**Affiliations:** 1. Basic Sciences in Infectious Diseases Research Center, Shiraz University of Medical Sciences, Shiraz, Iran; 2. Dept. of Parasitology and Mycology, School of Medicine, Shiraz University of Medical Sciences, Shiraz, Iran; 3. Dept. of Parasitology and Mycology, Faculty of Medicine, Zahedan University of Medical Sciences, Zahedan, Iran

**Keywords:** Wild boar, *Fasciola hepatica*, Iran

## Abstract

Wild boars may be infected with several zoonotic parasitic infections including *Fasciola* spp. We reported a case of *Fasciola* infection in a wild boar in Bushehr Province in southwestern Iran. The sample was isolated from the liver of a hunted wild boar. A few of adult worms were fixed and stained. DNA was extracted from apical and lateral parts of the worms and PCR amplified, targeting NADH dehydrogenase subunit 1 (nad1) and cytochrome C oxidase subunit 1 (cox1) mitochondrion genes. Although the worm was quite long and looked much similar to *F. gigantica*, sequencing and analysis of PCR products of nad1 and cox1 genes revealed that the isolate has the most similarity with *F. hepatica*. This is the first report of molecular evaluation of *Fasciola* spp. from wild boar in Iran.

## Introduction

Genus *Fasciola* (Platyhelminthes: Trematoda: Digenea) has a worldwide distribution and is considered as a liver fluke in a wide range of animals (1). *F. hepatica* and *F. gigantica* are two main species of parasites which infect both humans and animals. These two zoonotic flukes transmitted through water and food, contaminated with worm metacercaria (2).

Fascioliasis is one of the most important worm infections in some of tropical countries, with infection rates of up to 90% in animals (1, 2). In some parts of Africa, and in few of other countries, including Iran, human and animal infections are a major health problem (3–5). The most recently reported incidence rate of human infection by this parasite is about 17 million cases. The disease is emerging as one of the most important parasitic diseases in the northern part of Iran (4). Overlapped distributions of *F. hepatica* and *F. gigantica* in Asia and co-infection with the two species in buffalo in northern Iran have been reported (1, 6, 7).

Infection of wild boars with *Fasciola* spp. has been reported from different areas of the world (8–11). In Iran, *Fasciola* infection was revealed in 3.5% of wild boars in north of the country, but the isolates have not been genetically confirmed (11). So far, there has been no molecular study to determine the genotype of *Fasciola* isolated from wild boars in Iran and this is the first report of molecular evaluation of *Fasciola* isolated from wild boars in Iran. DNA sequencing of nad1 and cox1 mitochondrial genes is useful markers for determining the genotypes and interspecies variations of these two species of *Fasciola* (12–14).

Here we report a case of *Fasciola* in a wild boar in the southern province of Bushehr in Iran, determined by molecular method, targeting these two mitochondrion genes.

## Case Report

**Fig. 1:**
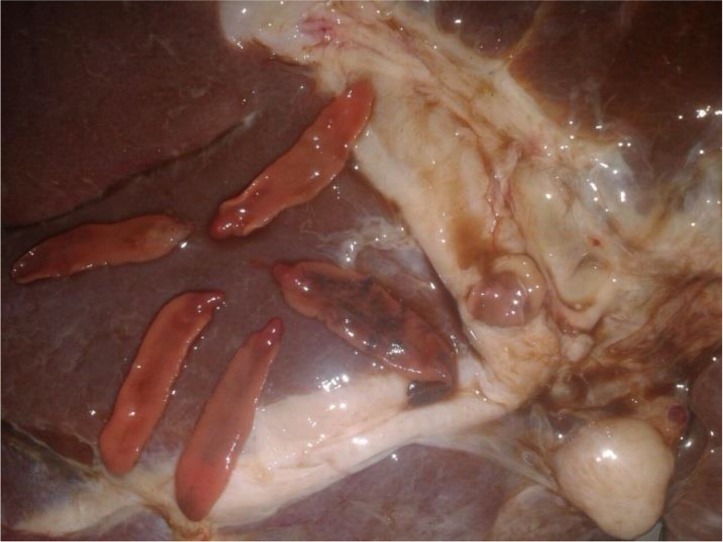
*Fasciola* adult worms in the liver of wild boar

**Fig. 2:**
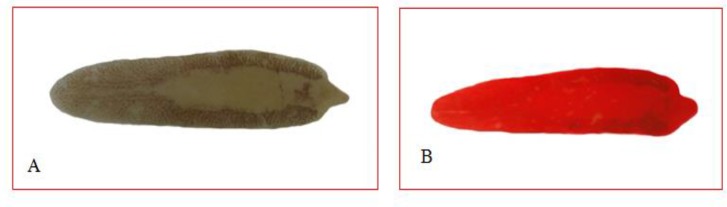
*Fasciola hepatica* isolated from the wild boar liver. A: unstained; B: stained with FAAL(Original)

*Fasciola* adult worms were isolated from the liver of a hunted wild boar, in Bushehr Province, southern Iran (Fig. 1). The grossly visible flukes were collected. The isolated worms were stored either in 70% alcohol, for molecular studies, or in 10% formalin for morphometric analysis. A few of samples were stained with FAAL (formalin, azocarmine, alcohol, and lactic acid) and were mounted by polyvinyl alcohol medium and photographed (Fig. 2). The isolated worms were similar and there was no much heterogeneity in their shape or sizes.

### Molecular evaluation

To extract the genomic DNA, apical and lateral parts of the adult worms were completely removed while the reproductive area was excluded. Specimens were completely crushed and then DNA was extracted manually, using the phenol-chloroform method. Extraction was performed using 15 μL of proteinase K and 300 μL of lysis buffer (50 ml of Tris-HCl (100 Mm), pH = 8; 1 mM of EDTA, pH= 8; 1% Tween 20). DNA was precipitated with 100% alcohol, re-suspended in 100 μL of distilled water, and stored at −20 °C until use. A 438 bp fragment from mitochondrial cox1 gene and a 535 bp fragment from mitochondrial nad1 genes were amplified by 5′–ACGTTGGATCATAAGCGTG-3′: forward and 5′-CCTCATCCAACATAACCTCT-3′: reverse primers.

PCR reaction was performed with a final volume of 25 mL containing 12.5 μL of master mix, 1 μL of each primer, 8.5 μL of distilled water and 2 μL of DNA. The thermocycler was programmed for PCR reaction as follows: an initial denaturation cycle at 94 °C for 2 min; 30 denaturation cycles at 94 °C for 1 min; annealing at 55 °C for 1.5 min; extension at 72 °C for 2 min; and final extension at 72 °C for 1 min. Amplicons were analyzed on a 1.5% agarose gel. DNAs from *F. hepatica* isolated from sheep in Iran, previously identified based on ITS1, ITS2, cox1, and nad1 genes, were included as positive controls in the PCR assays (13, 14). DNA was purified from the gel, using Vivantis DNA purification kit (Vivantis Technologies Sdn. Bhd. Selangor Darul Ehsan, Malaysia) based on the manufacturers’ instructions. PCR product was sequenced in both directions, with the same primers used for amplification, to determine the *Fasciola* species. Sequences were aligned, using CLCS Qiagen software, and compared with those of existing cox1 and nad1 reference sequences related to the genotypes of *Fasciola* spp. available in the GenBank.

### PCR-RFLP analysis

A PCR-RFLP method was used to specifically distinguish *Fasciola* species in ITS1 with *RsaI* restriction enzyme, as previously described (13).

**Fig. 3:**
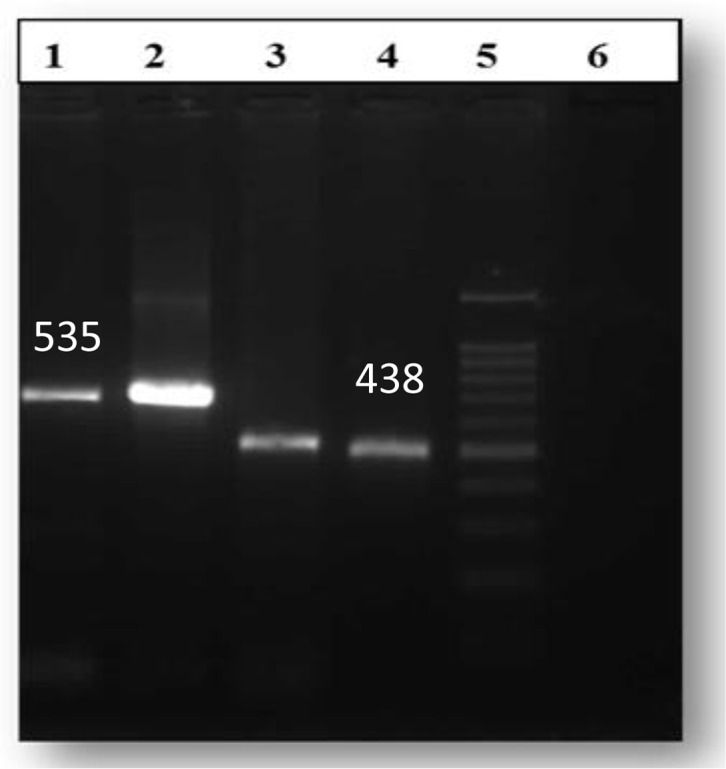
PCR-amplified mitochondria gene fragments of *Fasciola* spp. isolated from wild boar. Lane 1: nad1 gene of *Fasciola* isolated from the wild boar; lane 2, nad1 gene of *F. hepatica* isolated from the sheep (positive control); lane 3: cox1 gene of *Fasciola* isolated from the wild boar; lane 4: cox1 gene of *F. hepatica* isolated from the sheep (positive control); lane 5: molecular marker; lane 6 negative control

**Fig. 4:**
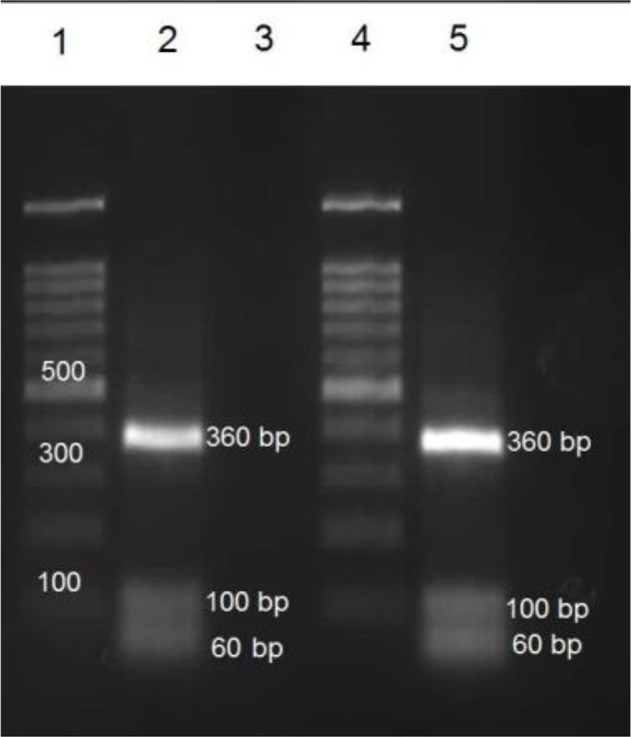
RFLP pattern of PCR products of *F. hepatica* digested by *RsaI* enzyme. Lane 1 and 4: molecular marker; Lane 2: *F. hepatica* (positive control) isolated from sheep liver (Fars Province); Lane 3: negative control; Lane 5: *F. hepatica* isolated from wild boar in the current study

The PCR result revealed a 438 bp fragment from mitochondrial cox1 gene and a 535 bp fragment from mitochondrial nad1 gene (Fig. 3). PCR-RFLP pattern with *RsaI* produced a 360, 100 and 60 bp fragments corresponding to *F. hepatica* (Fig. 4).

**Fig. 5:**
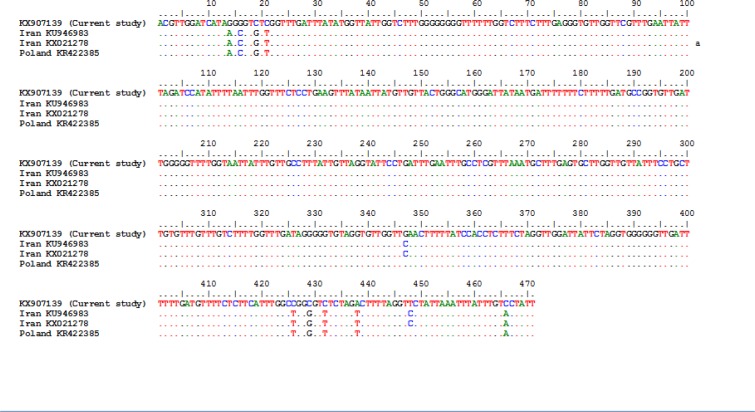
Alignment of sequences of the cox1 gene of *F. hepatica* isolated from wild boar in the southern state of Bushehr, Iran. KX907139: *F. hepatica* isolated from wild boar (current study); KU946983.1: *F. hepatica* reported from Iran; KX021278.1: *F. hepatica* isolated from cattle, Iran; KR422385.1: *F. hepatica* isolated from bison (Poland)

The sequence of cox1 isolate showed 97% identity with available sequences of *F. hepatica*, including KU946983, *F. hepatica* reported from Iran; and KX021278, *F. hepatica* isolated from cattle in Iran; KR422385, *F. hepatica* isolated from bison (Poland) (Fig. 5).

The sequence of nad1 of the isolate showed the most identity with available sequences of *F. hepatica* including KR422395.1 (from *Ovis aries* in Poland), AF216697.1 (from Australia), KR422396.1 (from *Ovis aries* in Poland), GQ356032.1 (from ovine in Iran), and GQ175362.1 (from Iran). The sequence data for the cox1 gene obtained in this study were deposited in GenBank with accession numbers of KX907139.

## Discussion

Fascioliasis is a foodborne neglected parasitic disease caused by *F. hepatica* and *F. gigantica*. In Europe, America, and Australia, only *F. hepatica* is a matter of concern while overlapping of both *F. hepatica* and *F. gigantica* has been observed in some regions of Africa and Asia, including Iran (6, 12). Wild boars may be infected with a few of parasitic infections including *Fasciola* species (8, 15–19). From the economic point of view, fascioliasis is important in sheep and cattle, but from the epidemiological perspective, the disease is important in other animals including the wild boars.

*Fasciola* may infect numerous domestic and wild animals, but susceptibility and pathology may be different based on the animal species (20). The host animal species strongly influence the phenotype of adult and also eggs of *Fasciola*. This is connected to the differences in size of the liver duct in the different hosts (21).

*Fasciola* infections in pigs, swine and wild boars have been reported from different areas of the world (8, 10, 11, 18, 22, 23). 1.3% of pigs in an area of Hunan Province in China were infected with *F. hepatica* (22). *F. hepatica* in wild boars has been reported in southern Germany (18). *F. hepatica* was reported in wild boars from Belorussian Polesie (24). In UK, *F. hepatica* has been reported in the bile duct of wild boars with increased diameter of bile ducts (10). Of 3021 livers of feral pigs examined in Sicily (Italy), 79 were regarded as having *F. hepatica* (23). *F. hepatica* infection was reported in 54 out of 3021 (4.37%) of a wild population of black pigs in Italy (23). A study about wild boar helminthic fauna in Tapada Nacional de Mafra (Portugal) revealed that 60.8% (14 out of 23 cases) of the animals were infected with *F. hepatica* (25).

In Iran, infection with *F. gigantica* has been reported in 2 out of 57 examined wild boars (11). The fluke species determined merely based on the morphometric features and no molecular evaluation has been performed on the isolates (11). In our previous study about helminth parasites of wild boars in Bushehr province, no case of *Fasciola* infection was detected in any of 25 studied wild boars (16).

The current study describes the genotype of *Fasciola* isolated from a wild boar, killed by local hunter in southwestern of Iran. This is the first report concerning the molecular evaluation of *Fasciola* isolated from wild boars in Iran.

In a study in Galicia (Spain), 40 out of 358 (11.2%) of wild boars have been parasitized by *F. hepatica* (8). Only 40% of the infected animals had *F. hepatica* eggs in their stools. Coprological detection of *F. hepatica* antigen revealed the coproantigens in 62.9% of the animals. By evaluating the percentage of hatching eggs, wild boars were considered as a very likely secondary reservoir for fascioliasis in that area. Morphometric analysis of the isolate revealed that *F. hepatica* in wild boar in that area has a normal development with its own characteristics. The gravidity and development of *F. hepatica* from wild boars were similar to those of sheep and cattle isolates (8).

Wild boars are very likely to have a role in the epidemiology of fascioliasis in area where these animals are infected with this fluke. Pigs may have a high transmission capacity of *Fasciola* to other animals or human. The metacercaria viability in pigs was similar to those of sheep and cattle isolates (20).

Pigs and donkeys were considered as the second reservoirs for fascioliasis in Bolivian Altiplano where the highest prevalence of human fascioliasis occurs (26). In fact, the normal fluke development in wild boars and shedding of viable *F. hepatica* eggs suggests that these animals may act as a secondary reservoir for *F. hepatica* (8, 20). These animals may contribute to the contamination of the environment through passing of parasite eggs.

Body length of 11.93–25.29 (17.01±3.43) and body width of 3.9–9.09 (7.03±1.19) were reported for *F. hepatica* isolated from wild boars (8). These parameters have been smaller in sheep and longer in cattle isolates. The maximum size reached by *F. hepatica* in wild boars has been bigger than that of sheep isolates (8). Wild boar bile duct is not unsuitable niche for development of *F. hepatica*. Pathological changes of fascioliasis in pigs does not include sever fibrous or calcification reaction (10). This allows the parasite to dilate the bile duct lumen and survive (23). Increase in the diameter of the bile duct was noticed in the infected boar in UK (10).

*F. hepatica* isolated from the wild boar in the current study was quite larger than those isolated from cattle and sheep in nearby areas in Iran. This is consistent with report which showed that *F. hepatica* from wild boars is larger than those isolated from sheep (8).

In the current study, molecular evaluation confirmed the species of the isolate as *F. hepatica*. This is the main species of *Fasciola* isolated from wild boars so far. Although the infection of wild boars was reported in Iran with *F. gigantica*, the species has not properly been confirmed since only morphometric analysis has been done and this approach is not appropriate for discrimination of these two species of *Fasciola* (13, 14).

## Conclusion

*F. hepatica* infection in wild boars might contribute to the contamination of the environment and have a considerable role in the epidemiology of fascioliasis in the region.
